# Supplementary material for: Iterative improvement in the automatic modular design of robot swarms
Source: PeerJ Comput Sci. 2020 Dec 7;6:e322. doi: 10.7717/peerj-cs.322 (PMC7924708; doi:10.7717/peerj-cs.322)
Supplement: Supplemental Information 3 [file peerj-cs-06-322-s003.zip › argos3/doc/api/standalone/a00422_source.html]

ARGoS: plugins/robots/eye-bot/control\_interface/ci\_eyebot\_proximity\_sensor.cpp Source File


- Main Page
- Related Pages
- Namespaces
- Classes
- Files

- File List
- File Members

# plugins/robots/eye-bot/control\_interface/ci\_eyebot\_proximity\_sensor.cpp

Go to the documentation of this file.

```
00001 
00007 #include "ci_eyebot_proximity_sensor.h"
00008 #include <argos3/core/utility/math/angles.h>
00009 
00010 #ifdef ARGOS_WITH_LUA
00011 #include <argos3/core/wrappers/lua/lua_utility.h>
00012 #endif
00013 
00014 namespace argos {
00015 
00016    /****************************************/
00017    /****************************************/
00018 
00019    static CRadians SPACING = CRadians(ARGOS_PI / 12.0f);
00020    static CRadians START_ANGLE = SPACING * 0.5f;
00021 
00022    /****************************************/
00023    /****************************************/
00024 
00025    CCI_EyeBotProximitySensor::CCI_EyeBotProximitySensor() :
00026       m_tReadings(24) {
00027       for(size_t i = 0; i < 24; ++i) {
00028          m_tReadings[i].Angle = START_ANGLE + i * SPACING;
00029          m_tReadings[i].Angle.SignedNormalize();
00030       }
00031    }
00032 
00033    /****************************************/
00034    /****************************************/
00035 
00036    const CCI_EyeBotProximitySensor::TReadings& CCI_EyeBotProximitySensor::GetReadings() const {
00037      return m_tReadings;
00038    }
00039 
00040    /****************************************/
00041    /****************************************/
00042 
00043 #ifdef ARGOS_WITH_LUA
00044    void CCI_EyeBotProximitySensor::CreateLuaState(lua_State* pt_lua_state) {
00045       CLuaUtility::OpenRobotStateTable(pt_lua_state, "proximity");
00046       for(size_t i = 0; i < GetReadings().size(); ++i) {
00047          CLuaUtility::StartTable(pt_lua_state, i+1                           );
00048          CLuaUtility::AddToTable(pt_lua_state, "angle",  m_tReadings[i].Angle);
00049          CLuaUtility::AddToTable(pt_lua_state, "value",  m_tReadings[i].Value);
00050          CLuaUtility::EndTable  (pt_lua_state                                );
00051       }
00052       CLuaUtility::CloseRobotStateTable(pt_lua_state);
00053    }
00054 #endif
00055 
00056    /****************************************/
00057    /****************************************/
00058 
00059 #ifdef ARGOS_WITH_LUA
00060    void CCI_EyeBotProximitySensor::ReadingsToLuaState(lua_State* pt_lua_state) {
00061       lua_getfield(pt_lua_state, -1, "proximity");
00062       for(size_t i = 0; i < GetReadings().size(); ++i) {
00063          lua_pushnumber(pt_lua_state, i+1                 );
00064          lua_gettable  (pt_lua_state, -2                  );
00065          lua_pushnumber(pt_lua_state, m_tReadings[i].Value);
00066          lua_setfield  (pt_lua_state, -2, "value"         );
00067          lua_pop(pt_lua_state, 1);
00068       }
00069       lua_pop(pt_lua_state, 1);
00070    }
00071 #endif
00072 
00073 
00074    /****************************************/
00075    /****************************************/
00076 
00077    std::ostream& operator<<(std::ostream& c_os,
00078                             const CCI_EyeBotProximitySensor::SReading& s_reading) {
00079       c_os << "Value=<" << s_reading.Value
00080            << ">, Angle=<" << s_reading.Angle << ">";
00081       return c_os;
00082    }
00083 
00084    /****************************************/
00085    /****************************************/
00086 
00087    std::ostream& operator<<(std::ostream& c_os,
00088                             const CCI_EyeBotProximitySensor::TReadings& t_readings) {
00089       if(! t_readings.empty()) {
00090          c_os << "{ " << t_readings[0].Value << " }";
00091          for(UInt32 i = 1; i < t_readings.size(); ++i) {
00092             c_os << " { " << t_readings[0].Value << " }";
00093          }
00094          c_os << std::endl;
00095       }
00096       return c_os;
00097    }
00098 
00099    /****************************************/
00100    /****************************************/
00101 
00102 }
```

---

Generated on 10 Jul 2018 for ARGoS by 
 1.6.1
